# Breast and Colorectal Cancer Screening and Sources of Cancer Information Among Older Women in the United States: Results From the 2003 Health Information National Trends Survey

**Published:** 2007-06-15

**Authors:** Steven S Coughlin, Zahava Berkowitz, Nikki A Hawkins, Florence Tangka

**Affiliations:** Epidemiology and Applied Research Branch, Division of Cancer Prevention and Control, National Center for Chronic Disease Prevention and Health Promotion, Centers for Disease Control and Prevention; Epidemiology and Applied Research Branch, Division of Cancer Prevention and Control, National Center for Chronic Disease Prevention and Health Promotion, Centers for Disease Control and Prevention, Atlanta, Ga; Epidemiology and Applied Research Branch, Division of Cancer Prevention and Control, National Center for Chronic Disease Prevention and Health Promotion, Centers for Disease Control and Prevention, Atlanta, Ga; Epidemiology and Applied Research Branch, Division of Cancer Prevention and Control, National Center for Chronic Disease Prevention and Health Promotion, Centers for Disease Control and Prevention, Atlanta, Ga

## Abstract

**Introduction:**

The number of people in the United States aged 65 years and older is increasing. Older people have a higher risk of dying from cancer; however, recent information about breast and colorectal cancer screening rates among women aged 65 years and older and about sources of health information consulted by these women is limited.

**Methods:**

We examined data from the Health Information National Trends Survey for women aged 65 years and older who had no personal history of breast or colorectal cancer. Women whose self-reported race and ethnicity was non-Hispanic white, non-Hispanic black, or Hispanic were included in the analysis. The overall response rate for the 2003 survey was 34.5%.

**Results:**

Women aged 75 years and older had lower rates of recent mammography (mammogram in previous 2 years) than did women aged 65 to 74 years. In both age groups, rates were especially low for Hispanic women and women with a household income of less than $15,000 per year. Rates of recent colorectal cancer screening (fecal occult blood test in previous year or endoscopy in previous 5 years) were markedly lower for non-Hispanic black women aged 75 years and older than for other women in this age group, and for Hispanic women aged 65 to 74 years than for non-Hispanic women in this age group. Screening rates were lowest for women with an annual household income of less than $15,000, no family history of cancer, no usual health care provider, or 1 or no provider visits in the previous year.

Differences were found in the groups' preferred channel for receiving health information. Women who had had a mammogram in the previous 2 years were more likely to pay attention to health information on the radio or in newspapers and magazines than were women who had not received a recent mammogram. Women who had had a recent colorectal cancer screening test were more likely to pay attention to health information in magazines or on the Internet than were those who had not. Personalized print and other publications were the most preferred channel for receiving health information.

**Conclusion:**

The results from this analysis suggest that educational materials about routine breast and colorectal cancer screening appropriate for women aged 65 years and older (especially low-income women, Hispanic women, and those aged 65 to 74 years) may be helpful.

## Introduction

People in the oldest age groups are of interest in cancer prevention and control because their numbers are increasing in the general U.S. population and older people have a higher risk of dying from cancer (1). Among women, about 58.3% of deaths from breast cancer and 78.5% of deaths from colorectal cancer occur in the group aged 65 years and older (2).

Nevertheless, medical and scientific authorities continue to debate the value of routine cancer screening for women in the oldest age categories. According to screening guidelines from the United States Preventive Services Task Force (USPSTF), the evidence that supports screening mammography every 1 to 2 years for women aged 40 years and older is generalizable to women aged 70 years and older if their life expectancy is not compromised by comorbid disease (3). However, uncertainty remains about whether the potential benefits of screening mammography outweigh the harms for older women (4-7), including increased anxiety and cost from possible false-positive test results and complications from diagnostic evaluation or biopsy procedures (3).

According to USPSTF guidelines for routine colorectal cancer screening among people aged 50 years and older, the age at which colorectal cancer screening should be discontinued is not known (8). Potential complications from endoscopy (flexible sigmoidoscopy or colonoscopy) include perforation, bleeding, and infection (9).

Little is known about older women's preferred channels and methods for seeking health information and how these relate to cancer screening practices (10-14). Studies show that women aged 65 years and older are less likely than younger women to undergo cancer screening (10,13). One explanation may be that older women are unaware of the recommendations for screening. Studies show that older people are less likely than younger people to use the Internet as a source of health information (15), but we do not know what information sources older women consult most often.

If lack of knowledge about screening recommendations accounts in part for lower screening rates among subgroups of older women (16,17), learning about their preferred information sources may help to guide future interventions and improve women's health.

To learn more about cancer screening practices and the use of medical information among older women, we analyzed data from the Health Information National Trends Survey (HINTS). Our objectives were to examine 1) breast and colorectal cancer screening practices among women aged 65 years and older, and 2) the sources of health information on cancer screening practices that these women sought. The research question tested in this study was whether older women who followed breast or colorectal cancer screening recommendations preferred different sources of health information from those who did not.

## Methods

### Survey sample

We obtained data from 3848 women who were interviewed from October 2002 through April 2003 as part of HINTS (18). The National Cancer Institute developed HINTS to produce data on the American public's need for, access to, and use of cancer-related information. The survey used a probability sample of telephone numbers in the United States to identify potential respondents. The HINTS interview was administered in either English or Spanish to a representative sample of adults aged 18 years and older of the noninstitutionalized civilian population. Hispanics and African Americans were oversampled. One person from each sampled household was selected for the interview after the household was screened. The response rate at the household-screening level was 55.0%, and the response rate for a completed interview by a sampled person was 62.8%, resulting in an overall response rate of 34.5%. HINTS collected information about the respondents' use of mass media, news media, and other information channels.

Our study sample was drawn from women aged 65 years and older who responded to the HINTS survey (n = 822 of 3848 female respondents of all ages). Of these, women whose self-reported race and ethnicity was non-Hispanic white, non-Hispanic black, or Hispanic were included in the analysis. We further refined the sample by excluding non-Hispanic women who reported that their race was neither white nor black (n = 24) because the numbers were too small for separate analysis, women whose race and ethnicity were reported as missing (n = 45), and women who had a personal history of breast or colorectal cancer (n = 78), leaving a study sample of 675 women.

### Study variables

The HINTS interview included questions about self-reported health status, demographic and socioeconomic characteristics, screening mammography, fecal occult blood testing (FOBT), and endoscopy. Each respondent was asked whether she had ever had a mammogram; participants who responded positively were then asked when they had received their last mammogram. Similar questions were asked about FOBT and endoscopy.

Other variables examined in the analysis related to media exposure and information seeking (e.g., hours of television or radio attended to per week, days respondents read newspapers or magazines in the previous week), attention paid to information about health or medical topics, whether or not respondents had looked for cancer information, where respondents had looked for cancer information, how much respondents relied on various channels of information, and Internet usage. Personalized print material was defined as reading material targeting specific lifestyles and family histories.

### Analysis

We analyzed data with SUDAAN, Version 9.1 (SAS Institute, Inc., Cary, NC), to account for the complex sampling of HINTS and its sampling weights, which were used in calculating population estimates and 95% confidence intervals (CIs). The age groups of interest were women aged 65 to 74 years and those aged 75 years and older. We used the chi-square test of independence to determine the association between adherence to screening and the covariates of media exposure and preferred channels of health information. Multivariate logistic analyses were performed to determine the effects of demographic and health coverage covariates on adherence to screening. We used pair-wise comparisons to test for significant differences between categories within each covariate.

## Results

About 85.0% of the women in our analysis were non-Hispanic white, 9.0% were non-Hispanic black, and 6.1% were Hispanic (Table 1). Approximately 16.9% of the women had a household income of less than $15,000 per year, and about 28.5% had less than a high school education. More than two-thirds (69.7%) had a family history of cancer. A large majority (85.9%) of the women had a usual health care provider. Almost all women (99.1%) had some type of health insurance (data not shown in table).

Data on the use of breast and colorectal cancer screening tests (Table 2) revealed that an estimated 89.4% of the women had ever had a mammogram, and 78.8% had had a recent mammogram (mammogram in the previous 2 years). Only 23.6% had had an FOBT in the previous year, and 38.8% had had endoscopy within the previous 5 years. About half (51.6%) had had an FOBT in the previous year or endoscopy in the previous 5 years.

Rates of recent mammography varied by age group, health history, and other demographic and socioeconomic factors (Table 3). Rates of recent mammography were lower for women aged 75 years and older than for those aged 65 to 74 years. Rates were much lower for Hispanic women in both age groups than for non-Hispanic women. Women in both age groups with a household income of less than $15,000 per year had low rates of recent mammography, as did those in both groups who were single (divorced/separated, widowed, or never married) and those who had 1 or no provider visits in the previous year. Older women who had no usual health care provider, especially those who were aged 75 years and older, also had low rates of recent mammography.

We also carried out a multivariate logistic analysis to see whether the associations between having a recent mammogram, income level, and having a usual health provider are confounded by having health insurance (data not shown). In a model for recent mammogram, which included age (65–74 vs 75–95 years), household income (<$15,000 vs ≥$15,000, or refused/don't know/missing), and usual provider, three variables — older age, lower income, and lack of a usual provider — were inversely associated with recent mammography (*P* < .03 in each instance). Health insurance coverage and education were not associated with recent mammography in this multivariate analysis (data not shown). However, almost all of the women had health insurance.

Rates of colorectal cancer screening varied by age group and other demographic factors, socioeconomic factors, and health history (Table 4). Non-Hispanic black women aged 75 years and older had the lowest rate of recent screening, and Hispanic women aged 65 to 74 years had lower rates than similarly aged non-Hispanic women. However, the numbers of women sampled in the respective categories were small. Rates of recent colorectal cancer testing were also low in each age group for women who had a household income of less than $15,000 per year, who were single, who had no family history of cancer, who had no usual health care provider, or who had 1 or no provider visits in the previous year.

In a multivariate model for colorectal cancer screening, the variables included age, education, household income, health care insurance, and a usual provider. Only lower educational attainment, lack of health insurance, and lack of a usual provider were significantly and inversely associated with colorectal cancer screening (*P* < .02 in each instance; data not shown). Household income was not significantly associated with colorectal cancer screening in multivariate analysis.

Data on media exposure indicate that television was the most common source of medical information for women in our analysis, regardless of whether they had recently had a mammogram or colorectal screening test (Table 5). Newspapers were the second most common source of medical information, and magazines were the third most common. Two percent of these older women did not attend to any type of media, 12% attended to one type, 16% to two types, and 70% to three or more types (data not shown in table). Women who had a recent mammogram were significantly more likely to pay attention to health information on the radio or in newspapers and magazines than were women who had not. Women who had received a recent colorectal cancer screening test were more likely to pay attention to health information in magazines or on the Internet than were women who had not.

Differences were also found between the groups' preferred channel for receiving health information (Table 5). Women who had a recent mammogram were significantly more likely to report that they wished to receive health information via personalized print, meeting with health care professionals, videocassette, audiocassette, CD-ROM, or other source than were women who had not. Women who had recently had a colorectal cancer screening test were significantly more likely to prefer receiving health information via personalized print materials or other publications (e.g., books, magazines) than were women who had not. Fewer than half of all women wanted to receive information via videocassette or audiocassette, e-mail or the Internet, or CD-ROM.

## Discussion

The results from this analysis of national survey data confirm and expand on results from prior studies of the use of cancer screening tests among older women (10-14,19-22), similar to the results reported by Cokkinides et al (20) using data from the Behavioral Risk Factor Surveillance System and by O'Malley et al (21) using Medicare data. The breast and colorectal cancer screening rates from the current study are slightly higher than those based on data from the National Health Interview Survey (NHIS) (22). The higher screening rates in HINTS may be explained by the use of household interviews to supplement telephone survey data in NHIS.

Culturally sensitive and appropriate educational materials about the value of screening mammography and colorectal cancer screening in reducing mortality from breast and colorectal cancer and about the potential benefits and harms of cancer screening may be helpful for older women, including low-income or minority women. Such materials should be available in both English and Spanish and should be modified to be relevant for older women from different ethnic and racial backgrounds. Educational materials should also be available for people with different levels of health literacy. Almost 29% of the women in our analysis reported having less than a high school education. Given that most health and medical information available to the public is written at a reading level higher than that of the average adult, this population may face challenges in accessing and understanding available printed health information (23-25).

Our finding that older women pay more attention to information disseminated on television and newspapers than through radio and the Internet is consistent with research suggesting that the Internet may not be the most efficient means for providing educational materials to older women (15). Our analysis also suggests that personalized print materials, other printed media, and telephone calls may be promising avenues for future efforts to disseminate information about cancer screening to older women who do not comply with screening recommendations.

Limitations of the present analysis include the cross-sectional design of the HINTS survey and the reliance on self-reported information about cancer screening and other variables. Questions for assessing cancer-related information on the HINTS questionnaire were, however, well-established and could be answered reliably and accurately by the adult population (26). New questions underwent cognitive testing (26). A further issue is that HINTS data did not allow us to distinguish between types of cancer in family histories. Other limitations include the possibility of bias due to nonresponse, given that the overall response rate in the 2003 HINTS was only 34.5%. Furthermore, the generalizability of the results to the overall U.S. population of women aged 65 years and older is unknown because of the low response rate and because noncivilian, institutionalized women and those without a household telephone were not included. Health insurance rates in the current study are higher, and cancer screening rates slightly higher, than those based on data from NHIS (22). The present study lacked information about comorbid health conditions or life expectancy, both of which may influence physician recommendations or decisions by older women about whether to undergo routine cancer screening. Finally, the results for black and Hispanic women are limited by the small number of nonwhite, non-Hispanic respondents.

Lack of knowledge about screening recommendations may partly account for lower screening rates among subgroups of older women. Our findings, therefore, that older women who adhere to breast or colorectal cancer screening recommendations and those who do not adhere to them prefer different sources of health information may help improve the health of women in this age group. Interventions designed to convey the potential benefits and risks of cancer screening to this group may benefit from knowledge of the sources of medical information used by older women and the channels of information preferred by them.

## Figures and Tables

**Table 1:** Demographic Characteristics and Health History Among U.S. Women Aged 65 Years and Older With No History of Breast or Colorectal Cancer (N = 675), Health Information National Trend Survey, 2003

Characteristics	Sample Size^a^	Pop. Est., % (95% CI)
**Age**		
65-74	368	58.3 (54.1-62.3)
75-95	307	41.7 (37.7-45.9)
**Race/ethnicity**		
Non-Hispanic white	568	85.0 (82.3-87.3)
Non-Hispanic black	60	9.0 (7.1-11.2)
Hispanic	47	6.1 (4.3-8.5)
**Household income**		
<$15,000	118	16.9 (14.0-20.4)
$15,000-<$25,000	177	29.3 (25.1-33.9)
≥$25,000	241	33.7 (29.7-37.9)
Refused/NA/DK/missing	139	20.1 (17.0-23.6)
**Education **		
<High school graduate	128	28.5 (25.7-31.5)
High school graduate	241	40.2 (38.0-42.3)
Some college	171	18.1 (16.3-20.0)
College graduate	133	13.2 (12.2-14.3)
**Marital status **		
Married/unmarried couple	232	47.2 (43.1-51.3)
Divorced/separated	82	9.6 (7.2-12.6)
Widowed	337	40.8 (36.5-45.2)
Never married	22	2.4 (1.4-4.1)
**Family history of cancer**		
Yes	454	69.7 (65.6-73.5)
No	216	30.3 (26.5-34.4)
**Self-rated health status**		
Excellent/very good	280	38.4 (34.5-42.5)
Good	224	35.3 (30.9-39.9)
Fair/poor	170	26.3 (22.3-30.7)
**Usual health provider**		
Yes	575	85.9 (82.5-88.7)
No	97	14.1 (11.3-17.5)
**Provider visits in previous year**		
≤1	120	17.1 (14.2-20.4)
2-4	301	44.9 (40.1-49.9)
≥5	244	37.9 (33.6-42.4)

CI indicates confidence interval; ref, refused; NA, not available; DK, don't know.

a Numbers may not equal 675 because respondents with "don't know" responses, refusals, or missing information were excluded.

**Table 2:** Screening Test Use by U.S. Women Aged 65 Years and Older With No History of Breast or Colorectal Cancer (N = 675), Health Information National Trend Survey, 2003

Screening Characteristic	Total Sample Size^a^	Adherence^b^ (n)	Pop. Est., % (95% CI)
Ever had mammogram	673	608	89.4 (85.5-92.4)
Mammogram within previous 2 years	669	528	78.8 (74.5-82.5)
Ever had fecal occult blood test (FOBT)	672	377	53.8 (48.9-58.6)
FOBT within previous year	662	166	23.6 (19.9-27.6)
Ever had sigmoidoscopy	655	194	26.2 (22.7-30.0)
Ever had colonoscopy	664	275	41.0 (36.3-45.9)
Sigmoidoscopy or colonoscopy within previous 5 years	661	272	38.8 (34.3-43.5)
FOBT in previous year or endoscopy in previous 5 years	660	350	51.6 (46.6-56.6)

CI indicates confidence interval.

a Numbers do not equal 675 because respondents with "don't know" responses, refusals, or missing information were excluded.

b Adherence is defined as mammography screening within previous 2 years, fecal occult blood test within previous year, or sigmoidoscopy or colonoscopy screening within previous 5 years.

**Table 3:** Recent^a^ Mammography Screening Among U.S. Women Aged 65 Years and Older With No History of Breast or Colorectal Cancer, by Age Group, Health Information National Trend Survey, 2003^b^

Characteristic	Age group 65-74 years		Age group ≥75 years	

	Total Sample Size	Pop. Est., % **(95% CI)**	Total Sample Size	Pop. Est., % **(95% CI)**
Total	367	83.0 (77.7-87.3)	302	72.8 (66.0-78.7)
**Race/ethnicity**				
Non-Hispanic white	298	83.1 (77.2-87.7)	265	72.5 (65.6-78.5)
Non-Hispanic black	38	94.2 (78.9-98.6)	21^c^	84.5 (54.2-96.2)
Hispanic	31^c^	66.4 (37.0-87.0)	16^c^	58.6 (24.1-86.3)
**Household income**				
<$15,000	52	55.9 (35.3-74.6)	66	63.9 (47.5-77.6)
$15,000-<$25,000	95	81.8 (70.0-89.6)	82	73.8 (61.1-83.4)
≥$25,000	151	93.7 (88.6-96.7)	90	74.8 (62.4-84.1)
Ref/NA/DK/missing	69	81.5 (67.5-90.3)	64	78.5 (61.9-89.2)
**Education**				
<High school graduate	59	72.2 (55.0-84.6)	68	69.6 (54.8-81.2)
High school graduate	141	87.0 (80.1-91.8)	99	70.3 (58.3-80.0)
Some college	91	85.8 (76.3-91.9)	78	79.0 (69.2-86.3)
College graduate	76	86.0 (73.0-93.3)	55	78.2 (60.8-89.2)
**Marital status**				
Married/unmarried couple	165	88.4 (79.7-93.6)	66	79.8 (65.7-89.1)
Divorced/separated	63	67.9 (45.6-84.2)	19^c^	66.8 (21.5-93.6)
Widowed	126	79.7 (69.4-87.1)	206	69.9 (61.1-77.4)
Never married	12^c^	71.2 (21.4-95.7)	10^c^	70.2 (16.2-96.6)
**Family history of cancer**				
Yes	258	85.3 (79.6-89.7)	194	74.5 (65.8-81.6)
No	105	75.9 (61.7-86.0)	107	70.5 (57.3-80.9)
**Self-rated health status**				
Excellent/very good	155	82.4 (74.2-88.4)	125	73.7 (61.0-83.4)
Good	121	85.8 (75.0-92.4)	101	74.7 (65.2-82.2)
Fair/poor	90	79.7 (67.1-88.3)	76	69.4 (54.7-81.0)
**Usual health provider**				
Yes	314	85.1 (78.7-89.9)	258	74.5 (66.7-80.9)
No	51	69.1 (50.7-82.9)	43	61.4 (41.6-78.1)
**Provider visits in previous year**				
≤1	72	79.3 (65.5-88.6)	44	49.4 (30.6-68.3)
2-4	162	79.8 (70.4-86.8)	138	75.2 (66.2-82.4)
≥5	132	88.9 (81.2-93.6)	112	78.3 (66.4-86.8)

CI indicates confidence interval; ref, refused; NA, not available; DK, don't know.

a In the previous 2 years.

b Totals exclude records with missing data.

c Numbers are too small for precise estimates.

**Table 4:** Prevalence of Recent Colorectal Screening^a^ Among U.S. Women Aged 65 Years and Older With No History of Breast or Colorectal Cancer, by Age Group, Health Information National Trend Survey, 2003

Characteristic	Age Group 65-74 years		Age Group ≥75 years	

	No. (Total Sample Size)^b^	Pop. Est., % **(95% CI)**	No. (Total Sample Size)^b^	Pop. Est., % **(95% CI)**
Total	362	52.4 (45.7-59.0)	298	50.5 (42.9-58.1)
**Race/ethnicity**				
Non-Hispanic white	295	53.2 (45.8-60.4)	261	52.0 (43.9-60.1)
Non-Hispanic black	38	58.9 (36.1-78.4)	21^b^	38.3 (15.8-67.2)
Hispanic	29^c^	33.3 (12.6-63.3)	16^c^	43.9 (16.0-76.2)
**Household income**				
<$15,000	50	38.2 (19.8-60.8)	66	45.7 (30.0-62.4)
$15,000-<$25,000	93	46.1 (33.6-59.1)	80	48.6 (34.0-63.5)
≥$25,000	151	59.1 (48.1-69.2)	88	54.2 (42.3-65.6)
Ref/NA/DK/missing	68	58.3 (43.1-72.1)	64	53.5 (38.6-67.9)
**Education**				
<High school graduate	58	44.2 (28.5-61.2)	66	36.4 (23.4-51.7)
High school graduate	140	52.9 (44.2-61.5)	97	54.4 (43.4-64.9)
Some college	89	58.2 (44.6-70.7)	78	51.6 (39.2-63.8)
College graduate	75	58.0 (44.4-70.5)	55	75.5 (58.1-87.3)
**Marital status**				
Married/unmarried couple	164	59.5 (49.6-68.7)	66	59.4 (46.1-71.5)
Divorced/separated	62	47.7 (30.0-66.1)	19^c^	43.8 (19.9-71.0)
Widowed	123	41.3 (31.6-51.9)	202	47.6 (38.0-57.4)
Never married	12^c^	29.0 (6.7-69.8)	10^c^	23.7 (3.1-75.2)
**Family history of cancer**				
Yes	255	54.5 (46.2-62.5)	190	53.5 (46.2-60.6)
No	104	47.4 (37.9-57.2)	107	45.8 (30.9-61.4)
**Self-rated health status**				
Excellent/Very good	153	45.9 (38.3-53.8)	122	47.0 (34.4-60.1)
Good	120	60.8 (47.1-73.0)	100	55.0 (43.2-66.3)
Fair/poor	89	49.7 (37.5-61.9)	76	50.1 (37.0-63.3)
**Usual health provider**				
Yes	309	54.7 (47.4-61.9)	255	52.6 (44.3-60.7)
No	51	38.2 (21.6-58.0)	42	35.9 (20.4-55.0)
**Provider visits in previous year**				
≤1	71	42.9 (27.8-59.5)	43	43.4 (24.5-64.4)
2-4	158	51.9 (40.3-63.4)	136	46.0 (35.1-57.2)
≥5	132	57.5 (47.7-66.7)	111	58.9 (47.9-69.1)

CI indicates confidence interval; ref, refused; NA, not available; DK, don't know.

a Fecal occult blood test within the previous year or sigmoidoscopy or colonoscopy within the previous 5 years.

b Totals exclude records with missing data.

c Numbers are too small for precise estimates.

**Table 5:** History of Media Exposure and Preferred Channel of Information, by Adherence^a^ to Cancer Screening Guidelines Among U.S. Women Aged 65 Years and Older With No History of Breast or Colorectal Cancer (N = 675), Health Information National Trend Survey, 2003

Characteristic	Mammography Screening					Colorectal Screening				

	Adhered		Did Not Adhere		***P*** ** value^b^**	Adhered		Did Not Adhere		***P*** ** value^b^**
	
	No.	Est. Pop., %(95% CI)	No.	Est. Pop., %(95% CI)		No.	Est. Pop., %(95% CI)	No.	Est. Pop., %(95% CI)	
**Media exposure**										
On TV	526	93.4 (90.3-95.6)	140	91.8 (85.2-95.6)	.57	348	94.1 (90.5-96.4)	309	91.6 (7.2-94.6)	.28
In newspaper	526	81.5 (77.5-85.0)	139	66.4 (57.6-74.1)	.002	348	81.8 (75.6-86.7)	307	75.3 (69.7-80.3)	.11
In magazines	527	74.3 (68.9-79.1)	140	53.5 (42.0-64.6)	.002	349	75.9 (68.2-82.2)	308	64.0 (57.2-70.4)	.02
On radio	524	46.6 (40.6-52.7)	141	35.4 (27.3-44.4)	.02	346	48.5 (41.3-55.7)	310	39.8 (31.7-48.5)	.11
On Internet	525	13.2 (10.5-16.5)	141	11.2 (6.4-18.9)	.57	350	16.3 (12.1-21.6)	307	9.3 (6.0-14.3)	.05
**Preferred channel of health information**										
Personalized print	518	80.1 (75.0-84.3)	136	65.8 (56.0-74.3)	.003	345	80.5 (74.8-85.1)	300	73.1 (65.8-79.2)	.05
Other publication	525	76.4 (71.4-80.9)	138	69.5 (60.9-76.9)	.12	346	78.5 (72.7-83.4)	308	71.2 (64.8-76.8)	.06
Meeting with health care professional	518	68.7 (64.8-72.5)	137	48.6 (38.6-58.6)	.000	343	63.8 (57.5-69.6)	303	64.9 (58.4-70.8)	.80
Telephone call	515	57.1 (51.1-62.8)	138	53.4 (42.6-63.9)	.49	340	55.4 (48.4-62.1)	305	56.9 (49.7-63.7)	.70
Videocassette	517	49.1 (43.4-54.7)	137	35.0 (24.8-46.7)	.02	342	46.5 (39.6-53.5)	303	45.9 (38.8-53.2)	.91
Audiocassette	521	41.5 (36.3-46.9)	141	31.0 (23.8-39.2)	.03	345	40.4 (33.8-47.2)	307	37.9 (31.1-45.3)	.64
E-mail/Internet	522	23.5 (19.6-28.0)	136	23.6 (15.6-34.1)	.99	347	24.4 (18.9-30.9)	302	23.0 (17.6-29.3)	.74
CD-ROM	520	23.2 (18.8-28.2)	140	11.6 (6.2- 20.6)	.01	345	21.0 (16.5-27.7)	306	20.0 (14.7-26.5)	.70
Another source^c^	521	8.6 (5.9-12.4)	140	4.3^d^ (1.8-10.1)	.1	345	9.4 (6.4-13.4)	307	5.4^d^(2.5-11.5)	.16

CI indicates confidence interval.

a Adherence is defined as mammography screening within previous 2 years, fecal occult blood test within previous year or sigmoidoscopy or colonoscopy screening within previous 5 years.

b *P* values for chi-square test of independence (analogous to the standard Pearson chi-square test for non-survey data).

c Responses to "another source" included the following: talking to people who had cancer; talking to family members, friends and other people; receiving information from cancer centers or societies; attending seminars and presentations; and watching television or listening to the radio.

d Number is too small for precise estimation.
