# Erratum, Vol. 4: Issue 3

**Published:** 2007-09-15

Because of an editing error, the footnotes in Table 4 of the article "Breast and Colorectal Cancer Screening and Sources of Cancer Information Among Older Women in the United States: Results from the 2003 Health Information National Trends Survey" were incorrect. The table should have three footnotes (a, b, and c). Footnote *a* should appear in the table title after "Prevalence of Colorectal Screening" and defines colorectal screening: FOBT within the past year or sigmoidoscopy or colonoscopy within the past 5 years. Footnote *b* appears in two column heads that read "n (total sample size)." The footnote says, "Totals exclude records with missing data." Footnote *c* appears in the table six times. Two occurrences are in the first column, headed "Age group 65-74 years" under the subhead "n (total sample size)," on rows labeled "Hispanic" and "Never married." Footnote *c* also appears in the column headed "Age group >75" under the subhead "n (total sample size)" on the rows labeled "Black," "Hispanic," "Divorced/separated," and "Never married."

Corrections were made on our Web site on July 18, 2007, and appear online at http://www.cdc.gov/pcd/issues/2007/jul/06_0104.htm. We regret any confusion this error may have caused.

